# Fibroblast Growth Factor 10 and Vertebrate Limb Development

**DOI:** 10.3389/fgene.2018.00705

**Published:** 2019-01-07

**Authors:** Libo Jin, Jin Wu, Saverio Bellusci, Jin-San Zhang

**Affiliations:** ^1^Institute of Life Sciences, Wenzhou University-Wenzhou Medical University Collaborative Innovation Center for Biomedicine, Wenzhou, China; ^2^Department of Pulmonary and Critical Care Medicine, The First Affiliated Hospital of Wenzhou Medical University, Wenzhou, China; ^3^Excellence Cluster Cardio-Pulmonary System, Universities of Giessen and Marburg Lung Center, member of the German Center for Lung Research, Justus-Liebig-University Giessen, Giessen, Germany; ^4^School of Pharmaceutical Sciences, Wenzhou Medical University, Wenzhou, China

**Keywords:** Fgf10, Limb, AER, β-catenin, Fgfr2b

## Abstract

Early limb development requires fibroblast growth factor (Fgf)-mediated coordination between growth and patterning to ensure the proper formation of a functional organ. The apical ectodermal ridge (AER) is a domain of thickened epithelium located at the distal edge of the limb bud that coordinates outgrowth along the proximodistal axis. Considerable amount of work has been done to elucidate the cellular and molecular mechanisms underlying induction, maintenance and regression of the AER. Fgf10, a paracrine Fgf that elicits its biological responses by activating the fibroblast growth factor receptor 2b (Fgfr2b), is crucial for governing proximal distal outgrowth as well as patterning and acts upstream of the known AER marker Fgf8. A transgenic mouse line allowing doxycycline-based inducible and ubiquitous expression of a soluble form of Fgfr2b has been extensively used to identify the role of Fgfr2b ligands at different time points during development. Overexpression of soluble Fgfr2b (sFgfr2b) post-AER induction leads to irreversible loss of cellular β-catenin organization and decreased Fgf8 expression in the AER. A similar approach has been carried out pre-AER induction. The observed limb phenotype is similar to the severe proximal truncations observed in human babies exposed to thalidomide, which has been proposed to block the Fgf10-AER-Fgf8 feedback loop. Novel insights on the role of Fgf10 signaling in limb formation pre- and post-AER induction are summarized in this review and will be integrated with possible future investigations on the role of Fgf10 throughout limb development.

## Introduction

Fibroblast Growth Factor 10 (Fgf10) is an evolutionary conserved secreted growth factor mediating mostly mesenchymal to epithelial signaling. Fgf10 belongs to the Fgf7 subfamily and shares similar biochemical and amino acid sequences with its constituent members (Fgf3, Fgf7 and Fgf22) (Min et al., 1998; Itoh and Ornitz, 2008).

The Fgf10 signaling cascade is initiated by its binding to epithelial Fgf receptors (Fgfrs) and heparin/heparan sulfate cofactor-proteoglycans (HS). Fgf10 mediates key intracellular signaling pathways in several cell types leading to the modulation of branching morphogenesis during development, wound healing and tissue repair (Itoh and Ohta, 2014). There are four known classical *Fgfr* genes (*Fgfr1-4*), these are alternatively spliced into “b” and “c” isoforms with exception to the *Fgfr4* gene. Alternative splicing confers tissue- plus cell- specific expression of these receptor isoforms and different affinities for ligands (Ornitz et al., 1996; Plotnikov et al., 2000). Fgf10 has been shown to bind with high affinity Fgfr1b and Fgfr2b compared to the other Fgf receptors (Ornitz et al., 1996; Ohuchi et al., 2000; Zhang et al., 2006). The Fgf10-Fgfr1b/Fgfr2b-HS signaling complex is essential for activating downstream signal transduction pathways, which include activation of Phosphoinositid-phospholipase C gamma (Plcγ), mitogen-activated protein kinases (Mapk), Protein kinase B (Akt) and signal transducer and activator of transcription proteins (Stat) cascades (Goetz and Mohammadi, 2013). For more information on this topic, please refer to a separate review published in this special issue on Fgf10 (Watson and Francavilla, 2018).

The developing vertebrate limb is a well-studied model to uncover the reciprocal cellular and molecular bases of harmonious organ growth and patterning (Allard and Tabin, 2009; Zeller, 2010). In mouse, limb development begins first with the induction of the forelimb buds at embryonic day 9.5 (E9.5) followed by the formation of the hindlimb buds at E10 on both flanks of the embryo (marking the future forelimbs and hindlimbs respectively) (Lu et al., 2008; Danopoulos et al., 2013). The early limb buds are composed of mesenchymal cells derived from the lateral plate mesoderm. The mesoderm induces the formation of a pseudostratified epithelium at the tip bud (Kieny, 1968; Saunders and Reuss, 1974), the so-called Apical ectodermal ridge (AER) (Todt and Fallon, 1984; Fernandez-Teran and Ros, 2008). It has also been shown that the skeletal and muscle elements of the forelimbs and the hindlimbs originate from the lateral plate mesoderm (Sun et al., 2002).

**Figure 1:**
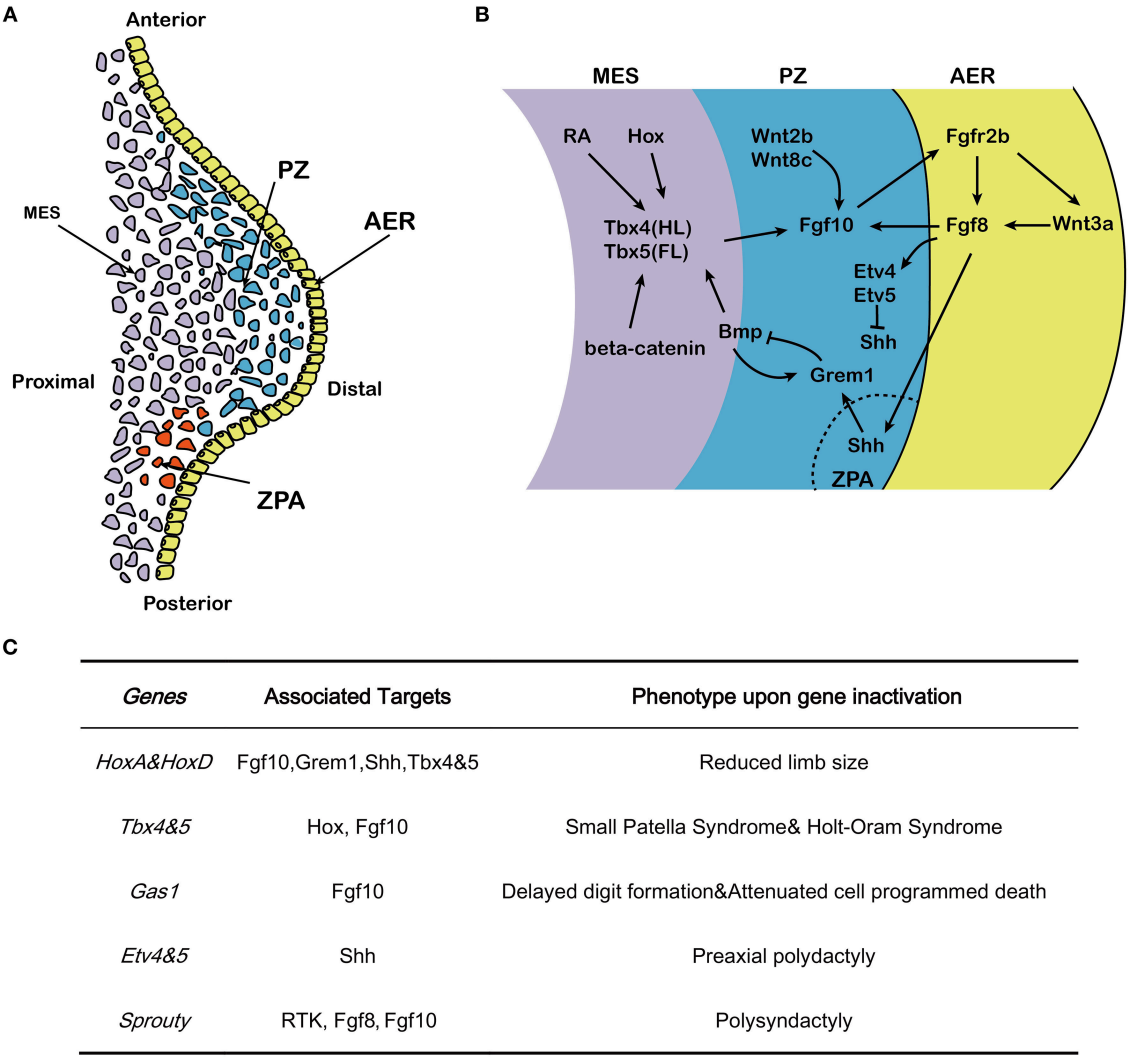
Effects of Fgf10 and its crosstalk network in early limb development. **(A)** Structure of early developing limb bud, from proximal to distal, there are three command centers: ZPA (controlling the anterior-posterior axis/digit identity), the AER and the PZ. Fgf10 expresses in the mesenchyme while Fgf8 expresses in the AER. **(B)** Hox/RA/β-catenin cooperatively triggers the activation of Tbx4/5 in the hindlimb and forelimb respectively. This leads to the up-regulation of Fgf10. Fgf10 activates Fgf8 in the overlying AER and they initiate a positive feedback loop that is essential for sustained limb growth. Shh, produced in the ZPA, acts to regulate correct anterior-posterior patterning during limb development. The Shh/Grem1/Fgf regulatory loop coordinates Shh signaling by the ZPA with Fgf signaling by the AER. **(C)** Specific genes correlated with Fgf10-AER-Fgf8 feedback loop and the targets as well as the effects of ectopic expression in limb development. MES, mesenchyme; PZ, progress zone; ZPA, zone of polarizing activity; AER, apical ectodermal ridge; HL, hindlimb; FL, forelimb.

The developing limb has three command centers: the AER, the zone of polarizing activity (ZPA) and the progress zone (PZ) (Figure 1A). Reciprocal interactions between the PZ and AER control the growth of the limb along the proximal-distal axis (Hara et al., 1998). After induction of the AER in the prospective limb field and outgrowth of the corresponding bud, the limb contains three distinct domains: the stylopod (humerus/femur), the zygopod (radius/tibia and ulna/fibula) and the autopod (carpal/tarsal, metacarpal/metatarsal, phalanges) (Saiz-Lopez et al., 2015). Genetic ablation of *Fgf10* in early mouse development results in death at birth and is associated with impressive developmental defects in multiple organs and tissues including the lung and the limb (Min et al., 1998; Sekine et al., 1999). In *Fgf10* knock out (KO) mice, limb bud formation is initiated but no further limb outgrowth is discernible, resulting in acute limb truncation with only rudimentary scapulae and pelvis remaining. In addition, skeletal staining at E17.5 in *Fgf10* KO fetuses confirms the absence of proximal limb elements such as the humerus (Min et al., 1998; Sekine et al., 1999). Notably, *Fgf10* KO mice display similar phenotypes to *Fgfr2b* KO mice in organogenesis (De Moerlooze et al., 2000) suggesting that, *in vivo*, Fgf10 acts mostly through Fgfr2b to control organogenesis. This review aims to provide an overview on the role and mechanism of Fgf10 signaling in limb development with a focus on the genetic data gathered from murine studies.

## Fgf10 Signaling Controls Limb Development

It has been previously described through tissue graft experiments that secreted factors from the limb mesenchyme are capable of initiating vertebrate limb bud formation (Saunders and Reuss, 1974). Fgf-soaked beads as well as cell aggregates expressing Fgf implanted in the flank of chick embryos led to the formation of ectopic limbs. Several Fgfs, including Fgf1, 2, 4, 8 and 10 were successfully tested (Cohn et al., 1995; Ohuchi et al., 1995, 1997; Crossley et al., 1996; Vogel et al., 1996). Interestingly, Fgf8 and Fgf10 are the only Fgfs expressed in the limb at the time of AER formation (Crossley et al., 1996; Vogel et al., 1996; Ohuchi et al., 1997). Both Fgf8 and Fgf10 are expressed before AER induction in the intermediate mesoderm and the lateral plate mesoderm within the limb field of the chick embryos respectively. Fgf8 and Fgf10 are also found in the AER and the PZ respectively (Crossley et al., 1996; Vogel et al., 1996). Similar expression pattern for *Fgf8* and *Fgf10* were observed in mouse limb buds at the time of AER induction (Min et al., 1998; Sekine et al., 1999). The precise expression of these Fgfs in regards to limb development before AER induction in mouse is still unclear. However, the study of Gros and Tabin (2014) using mouse limbs from *Fgf10* null embryos clearly shows that Fgf10 participates in the regulation of epithelial mesenchymal transition of the somatopleure (see An “untold story” is emerging for Fgf10 signaling in the pre-AER phase).

During early development, the position of the forelimb and hindlimb along the cranial-caudal axis is controlled by *Hox* genes (Pineault and Wellik, 2014). Disruption of *HoxA* and *HoxD* genes results in seriously reduced limb size, which was initially associated with sonic hedhehog (Shh) downregulation (Figure 1C) (Crossley et al., 1996). Early-activated homeobox gene A (*HoxA*) and homeobox gene D (*HoxD*) stimulate expression of *Fgf10* and result in considerable expression of *Fgf8* in the AER. *Hox* also regulate expression of *gremlin1* (*Grem1*) and Shh and hence maintain the cross-talk among Shh, Gli3, HoxA and HoxD (Zakany et al., 2007). Hox also induce *T-box 5* (*Tbx5*) in the forelimb and *T-box 4* (*Tbx4*) in the hindlimb (Pineault and Wellik, 2014) (Figure 1B). The differential expression of the two paralogous transcription factors Tbx4 and Tbx5 serve as molecular evidence for determining early limb identity (Gibson-Brown et al., 1996; Minguillon et al., 2009). In human, *TBX5* mutation causes Holt–Oram syndrome (HOS) which is associaited with upper limb abnormalities (Basson et al., 1997; Bongers et al., 2004), while *TBX4* mutation results in small patella syndrome, which is characterized by foot dysplasia (Bongers et al., 2004) (Figure 1C). Inactivation of *Tbx4* or *Tbx5* in mice leads to lack of *Fgf10* expression in the lateral plate mesoderm where the forelimb or hindlimb would normally form, respectively (Rodriguez-Esteban et al., 1999). During limb bud formation in mouse, both transcription factors trigger *Fgf10* expression in the limb mesenchyme (Saunders and Reuss, 1974; Vogel et al., 1996; Naiche and Papaioannou, 2003; Minguillon et al., 2005). Fgf10 signaling then triggers Fgf8 expression. Furthermore, genetic ablation of either *Tbx4* and *Tbx5* results in outgrowth limb bud defects (Saunders and Reuss, 1974; Vogel et al., 1996; Ng et al., 2002; Naiche and Papaioannou, 2003; Duboc and Logan, 2011).

The AER, which is histologically characterized by a local thickening of the ectoderm, is the earliest signaling domain to be induced during limb bud formation. It is initiated from the lateral plate mesoderm and is the result of mesenchymal-Fgf10/AER-Fgfr2b signaling (Sekine et al., 1999; De Moerlooze et al., 2000). Using chick and zebrafish embryos, it was shown that Fgf10 expression in the proliferating cells of the PZ is stabilized by Wnt2b and Wnt8c in the forelimb and hindlimb, respectively (Kawakami et al., 2001; Ng et al., 2002), and induces the expression of *Wnt3a* and *Fgf8* in the AER (Figure 1B). *Fgf8* expression, which is stabilized by Wnt3a (Kengaku et al., 1998; Kawakami et al., 2001), in turn acts on the underlying mesenchymal cells located in the PZ to maintain *Fgf10* expression and triggers the amplification of the different skeletal progenitors of the limb (Mariani et al., 2008) [for review see (Fernandez-Teran and Ros, 2008)] (Figure 1B). Therefore, Fgf10 and Fgf8 display completementary roles in the AER, and interact in a feed-forward regulation loop (Ohuchi et al., 1997; Kawakami et al., 2001). The Fgf10-AER-Fgf8 feed-forward loop is integrated with other signaling pathways, thereby constituting an elaborated network of interactions to govern limb formation.

Ets variant 1 (Etv1), a member of the E26 transformation-specific (Ets) transcription factors and transcriptional co-activator Ewings sarcoma RNA binding protein 1 (Ewsr1) (Munchberg and Steinbeisser, 1999; Park et al., 2013), acts downstream of AER-Fgfs to maintain a high level of *Fgf10* expression in the AER mesenchyme by stimulating the *Fgf10* promoter in a collaborative way (Yamamoto-Shiraishi et al., 2014). In addition, growth arrest specific gene 1 (*Gas1*) expression in the mesenchyme preserves Fgf10 expression at high level while Fgf10 is of vital importance in maintaining the expression of Fgf8 in AER. *Gas1* KO mice display delayed digit formation, reduced cell proliferation in AER and distal mesenchyme as well as attenuated cell programmed death in interdigital cells (Liu et al., 2002). The *Twist* gene encoding a basic helix–loop–helix (bHlh) transcription factor (Simpson, 1983; Jurgens et al., 1984) is expressed in the lateral plate mesoderm before limb bud formation (Stoetzel et al., 1995). *Twist* KO embryos display reduced expression of *Fgf10* in the limb bud mesenchyme, and is associated with decreased bud growth from its initiation onward (O'Rourke et al., 2002). Etv4 and Etv5 are two additional members of the Ets family of transcription factors working downstream of Fgf signaling and are both expressed in the limb mesenchyme (O'Rourke et al., 2002). In mice, *Etv4/Etv5* double KO embryos display ectopic expression of *Shh*, which in turn leads to preaxial polydactyly. Therefore, Etv4 and Etv5, working downstream of AER-Fgf, function to repress *Shh* expression outside of the ZPA (Zhang et al., 2009).

The AER activity is also mediated by other Fgfs emanating from the AER and acting on the underlying mesenchyme. Fgf8 expression in the AER is detected before the other Fgfs and spans the entire AER. Deletion of *Fgf8* in mice leads to decreased amplification of the mesenchymal progenitors resulting in impaired limb development (Lewandoski et al., 2000). Concomitant overexpression of *Fgf4* in AER cells where *Fgf8* has been deleted allows the rescue of limb development demonstrating that Fgf4 can functionally replace Fgf8 and that the *Fgf8* KO limb phenotype is likely the consequence of the unique timing of *Fgf8* expression vs. the other Fgfs (Lu et al., 2006).

The role of Sprouty (Spry) proteins, which were first explored in drosophila, have also been implicated in the negative regulation of Fgf10 signaling in other organs such as the lung (Minowada et al., 1999). Spry proteins are negative regulators of receptor tyrosine kinase (Rtk)-mediated Map kinase signaling (Hacohen et al., 1998). In a transcriptome analysis of early proximo-distal patterning of the Xenopus laevis limb bud, correlations of early gene expression patterns between Spry1, 2 and 4 and the expected range of Fgf8 and Fgf10 signaling in the developing limb bud has prompted a role for Spry in regulating Fgf signaling in normal limb development. It has been proposed that Spry2 acts as a negative regulator of AER-Fgf8 [(Lewandoski et al., 2000; Impagnatiello et al., 2001); Figure 1C]. Although the corresponding Spry roles in mammals are still scarce, *Spry4* KO mice displayed polysyndactyly, which is delineated by fusion and duplication of digits at the forelimbs. A large mutagenesis screening has identified the *Spry4* gene as a candidate regulator of normal limb formation (Taniguchi et al., 2007).

## Fgfr2b Signaling in Post-AER Induction

As stated above, Fgf10 shows high affinity to Fgfr2b. Fgfr2b has also been independently investigated for its role in limb genesis. Inactivation of *Fgfr2b* in the embryo leads to limb agenesis (De Moerlooze et al., 2000). RNA interference combined with Cre-LoxP system was carried out to attenuate *Fgfr2b* expression in the PZ of the limb and caused dysmorphia of digits (Bellusci et al., 1997). More recently, conditional inactivation of *Fgfr2* in the AER was carried out using the *Msx2-Cre* driver line to target the limb ectoderm (Lu et al., 2008; Yu and Ornitz, 2008). Consequently, *Fgfr2*^*Msx*2−*Cre*^ embryos displayed complete hindlimb agenesis. The forelimb had normal stylopod (humerus/femur) and zygopod (radius/tibia and ulna/fibula) but absent autopod (carpal/tarsal, metacarpal/metatarsal, phalanges) (Yu and Ornitz, 2008). This demonstrated that inactivation of *Fgfr2* in the epithelium of the limb post-AER induction leads to the genetic ablation of the AER. In addition, the loss of autopod is consistent with AER inhibition in the forelimb (illustrated by decreased AER-Fgf8 expression). These results have been validated using a double transgenic system allowing the ubiquitous and robust expression of a secreted form of Fgfr2b capable of sequestering the endogenous Fgfr2b ligands at different time points during or post-AER induction (Danopoulos et al., 2013).

## Fgfr2b Ligands and Canonical β-catenin Signaling

One of the major mechanistic insight into Fgfr2b signaling is the rapid inhibition of canonical Wnt signaling following Fgfr2b ligand inhibition. Using the Topgal reporter allele, a complete decrease in Wnt signaling was reported 1 h after doxycycline-intraperitoneal injection (Dox-IP). Such a decrease was validated by detecting the nuclear phosphorylated form of β-catenin (Danopoulos et al., 2013). How Fgfr2b signaling impacts β-catenin signaling is still unclear. *In vivo*, some of the genes were down-regulated upon inhibition of Fgfr2b ligands (6 h post Dox-IP at E11.5) activity including Wnt ligands (*Wnt3a, Wnt3, Wnt7a, Wnt7b, Wnt16*), Wnt receptors (*Fzd4, Fzd8, Fzd9*) and secreted Wnt inhibitor (*Wif1*) as well as Wnt1-induced secreted protein 1 (*Wilsp1*) (Danopoulos et al., 2013). Genes up-regulated include *Frzb*, a gene encoding a Wnt binding protein acting as a competitor for the Wnt receptor *Frzd* as well as *Pitx2*, encoding a transcription factor interacting with β-catenin and *Dkk1*, a gene encoding a secreted Wnt ligand inhibitor (Danopoulos et al., 2013). The collective regulation of these genes is likely to impact canonical Wnt signaling.

Immunofluorescence staining for β-catenin in the AER of experimental and control E11.5 limb 1 h after Dox-IP revealed reduced expression level and cellular disorganization of β-catenin upon Fgfr2b ligand inhibition (Danopoulos et al., 2013). With less β-catenin available, this could be sufficient to lead to decreased Wnt signaling. In zebrafish, somitic mesoderm-derived retinoic acid (RA) signaling resulted in activation of *Wnt2b* expression in the mid-mesoderm, that signals to trigger *Tbx5* expression, In turn, Tbx5 is required for Fgf signaling in the limb bud and then brings about the activation of PR domain containing 1 (*Prdm1*), which then stimulates *Fgf10* expression (Mercader et al., 2006). The same series of events are likely occuring in the developing mammalian limbs since *Prdm1* expression is conserved between zebrafish and tetrapods. More studies will have to be done in the future to elucidate mechanistically the impact of Fgf signaling on β-catenin expression level and localization.

In order to elucidate the impact of transient inhibition of Fgfr2b signaling in limb development, the *Rosa26*^*rtTA*^ mice were crossed with *Tg(tet(O)solubleFgfr2b (Tg)* transgenic mice to generate double transgenic mice [*Rosa26*^*rtTA*/+^*;Tg/*+], called hereafter DTG mice. These DTG mice were crossed together to generate experimental DTG and control single transgenic (STG) [*Rosa26*^*rtTA*/+^*;* +*/*+] embryos. Allelic series for DTG embryos (with one or two copies of *Rosa26*^*rtTA*^ and one or two copies of *Tg*) were generated. This allowed analyzing the impact of different levels of soluble Fgfr2b on limb development. Pregnant mice carrying both control STG and experimental DTG embryos were injected intraperitoneally with a single dose of doxycline at E7.5, E8.5, E9.5 or E10 (Parsa et al., 2008, 2010). The resulting impact on the formation of the cartilage and bone in the limb was analyzed at E18.5 following alcian blue/alizarin red staining. Dox-IP at E7.5, 2 days before AER induction, indicated no phenotypic differences in the limbs of STG and DTG embryos. Dox-IP at E9.5, at the time of forelimb AER induction and 12 h before hindlimb AER induction led to complete forelimb and hindlimb agenesis supporting the previously reported *Fgfr2b* and *Fgf10* KO phenotypes. The difference in phenotype between E9.5 (limb agenesis) and E7.5 (no limb defects) demonstrated that sFgfr2b expression in our model is indeed reversible following Dox-IP injection (Danopoulos et al., 2013). The phenotype resulting from a single Dox-IP at E8.5 is described in the paragraph below.

## A Potential Connection Between Thalidomide and Fgf10 Signaling

It has been proposed that the mesenchymal progenitors for the three skeletal domains (mostly for the stylopod) are being already amplified in a Fgfr2b ligand-dependent fashion during pre-bud formation from E8.5-E9.5 (Danopoulos et al., 2013; Gros and Tabin, 2014). E18.5 DTG heterozygous embryos ([*R26*^*rtTA*/+^*;Tg/*+]) resulting from Dox-IP at E8.5 (corresponding to 1–1.5 days before forelimb and hindlimb induction, respectively) showed shorter forelimbs and almost normal hindlimbs. Bone/cartilage staining indicated normal scapula but reduced humerus as well as radius and ulna. A phenotypic difference between the right and left limbs was also observed. The right limbs were more severely affected, with a near absence of humerus and shortened femur, and the complete absence of ulna and fibula. Furthermore, while digits had formed in both forelimbs and hindimbs, these digits were fewer on the right side limbs. In addition, DTG homozygous embryos [*R26*^*rtTA*/*rtTA*^*;Tg/Tg*] display forelimb agenesis and had severely reduced hindlimbs. Bone/cartilage staining revealed a shorter femur and no elements beyond a rudimentary tibia. Once again, the right hindlimbs appeared more severely affected than the left hindlimbs. The reason for this difference is still unclear and will deserve further investigation (Danopoulos et al., 2013).

The limb phenotype displayed upon inhibition of Fgfr2b signaling at E8.5 is similar to the severe proximal truncations observed in human babies exposed to thalidomide, a drug which became popular in the late 1950s (Cohen, 1962). It was initially prescribed for its effect on insomnia, anxiety, gastritis, tension and against nausea to alleviate morning sickness in pregnant women. Thalidomide adminstration to pregnant woman has been shown to cause phocomelia, a condition that involves malformations of the arms and legs. Short arm bones, fused fingers and missing thumbs as well as absent pelvic bones often occur (Kim and Scialli, 2011). The expression of nuclear factor k-B (Nf-κB), an important factor in mediating limb development, is drastically weakened upon thalidomide treatment, which in turn blocks limb cells to express Fgf10 and Twist in the PZ mesenchyme, followed with attenuated expression of Fgf8 in the AER. This process destroys the Fgf10/Fgf8 feedback loop between the PZ and AER and consequently prevents limb initiation (Crossley et al., 1996; Bushdid et al., 2001). It has been proposed that thalidomide limb teratogenicity is linked to oxidative stress damage, DNA intercalation and inhibition of angiogenesis. Cereblon has been identified as primary target of thalidomide and forms a E3 ubiquitin ligase complex with damaged DNA binding protein 1 and Cullin4a (Groisman et al., 2003; Ohtake et al., 2007). Damaged DNA binding protein 1 (*Dbb1*) and cullin 4a (*Cul4a*) regulate the expression of Fgf8 and limb development. Inactivation of cereblon (*Crbn*) in zebrafish leads to defective fin and otic vesicle development (Ito et al., 2010). It is likely that the mechanisms mentioned above can operate in parallel to impair limb formation. It will be important to better define the mechanisms of action of thalidomide on Fgfr2b signaling during this early phase of limb development in future studies.

## An “Untold Story” Is Emerging for Fgf10 Signaling in the Pre-AER Phase

**Figure 2:**
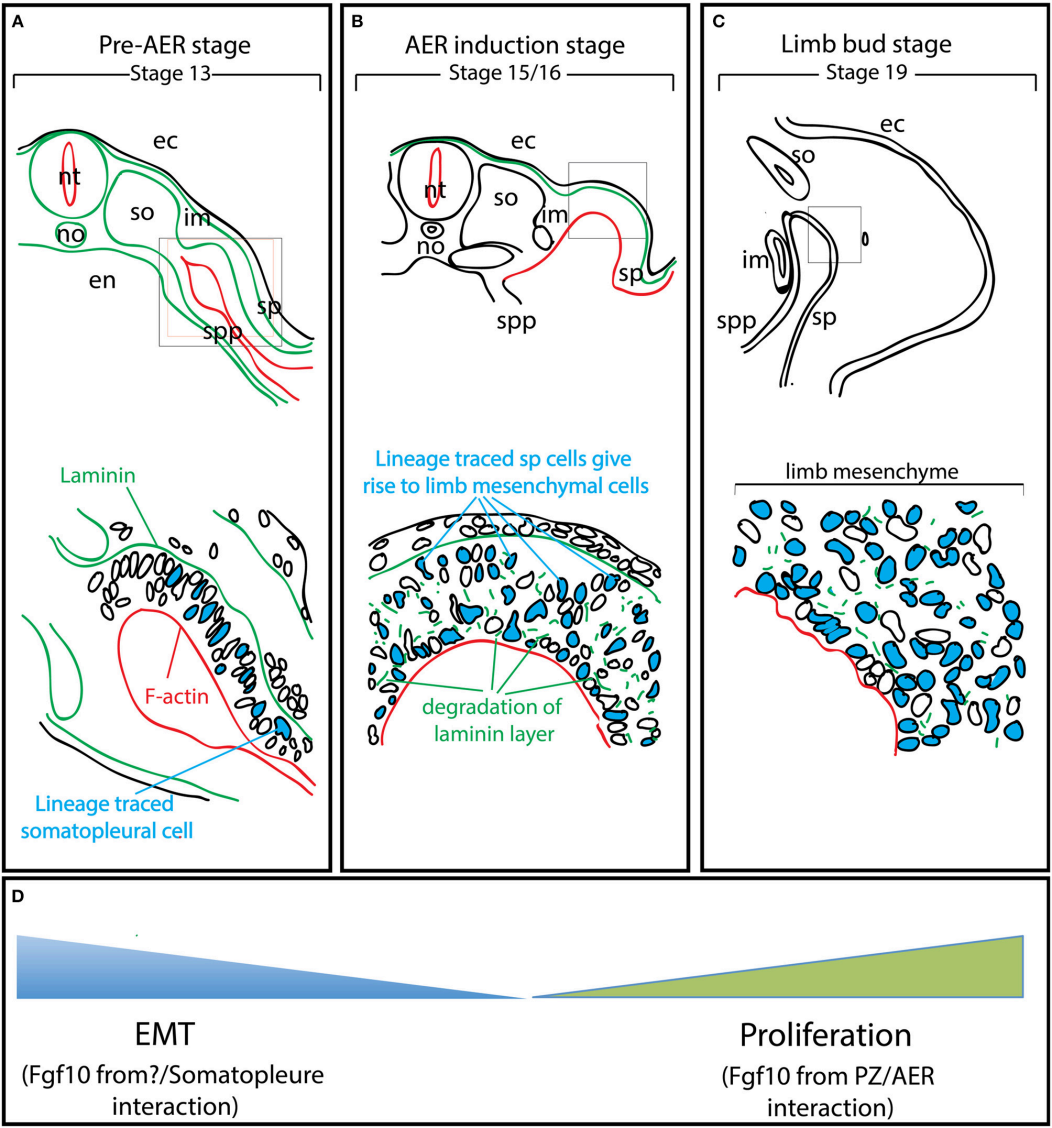
EMT from the somatopleure epithelium is at the heart of limb induction. Adapted from Gros and Tabin's paper (Gros and Tabin, 2014). **(A)** Lineage labeling of the cells in the somatopleure. Note that the cells express laminin on their basal side and F-actin on their apical side. **(B)** The labeled cells undergo EMT. Note that this EMT is associated with the degradation of the laminin layer on the basal side. **(C)** Most of the mesenchymal cells in the rudimentary limb bud post-AER induction (Stage 19) arise from EMT. **(D)** The mesenchymal limb progenitors are first formed through EMT up to the AER induction stage and then amplified post-AER induction through the interaction of the Progress zone (Fgf10-positive) and the Apical Ectodermal ridge (AER). ec, ectoderm; en, endoderm; im, intermediate mesoderm; no, notochord; nt, neural tube; so, somites; sp, somatopleure; spp, splanchnopleure.

Gros and Tabin reported a new function for Fgf10 during pre-AER induction, namely, the induction of epithelial to mesenchymal transition (EMT) from the somatopleural epithelium to form the early mesenchymal limb progenitors, the building blocks of the future limbs (Gros and Tabin, 2014). It has been described in chick model that at stage 13–14 (Figure 2A), the somateuploral lateral plate mesoderm of the limb field which starts out as an epithelial-like structure will ultimately generate the progenitors for the limb bud mesenchyme through a process ressembling the EMT (Figure 2D). However, this is not likely a classical EMT as the characteristic transcription factors, *Snail* and *Slug* are not expressed. In addition, the somatopleural “epithelium,” which is mesoderm-derived is unlikely to be a true epithelium. The role of Fgf10 on the somatopleural “epithelium” is therefore far from being clear and deserves further investigations. Lineage tracing using a GFP reporter in the somatopleure of stage 13-14 chick embryos demonstrated that most, if not all, mesenchymal cells of the early limb bud (stage 15-16, time of AER induction for the forelimb) (Figure 2B) originate from the “epithelial” somatopleure. Later on, induction of several Fgfs at the level of the prospective AER will allow the establishment of a feedback loop that will amplify the mesodermal progenitors and allow the formation of the different limb segments along the proximal-distal axis. It has also been reported that ectopic gain of function of Fgf10 (up to stage 17) induces limb bud formation (Figure 2C). Gros et al. show that this occurs through EMT from the epithelial trunk somatopleural cells but not from the amplification/proliferation of mesenchymal cells of the same rostrocaudal level (Gros and Tabin, 2014). In addition, failure of ectopic Fgf10 signaling (beyond stage 17) to induce limb formation was thought to be due to the trunk mesenchyme which became determined and was no longer capable to be redirected to a limb fate: this fundamental hypothesis, almost a dogma in the lung field, is no longer valid.

## Fgf10 Signaling in Mesenchymal Progenitor Formation

The proposed molecular mechanism taking place during the pre-AER phase, based on the work from the Tabin's group, is that Fgf10 acts on the epithelial somatopleure to induce EMT (Gros and Tabin, 2014). This allows the formation of the early mesenchymal limb progenitors that will proliferate and differentiate during the post-AER phase to form the different limb segments. It also has been demonstrated that Fgfr2b ligand(s) signaling are critical in pre-AER induction (E8.5-E9.5) stage to allow the formation of the early mesenchymal progenitors for the limb. Future experiments should be designed to answer the following questions: Where and when is Fgf10 signaling active in the limb field before the induction of the AER (E8.5-E9.5)? Whether EMT is a direct effect of Fgf10 on the somatopleure epithelium should also be evaluated as well as the consequences of different Fgf10 mediated-pre-AER mesenchymal progenitor pool sizes on limb development. Finally, what are the different lineages that Fgf10-positive cells in the pre-AER pool contribute to?

## Author Contributions

SB and J-SZ conceived this paper. LJ and JW wrote the manuscript. SB, J-SZ and JW designed and drew the figures. LJ, J-SZ, and SB edited the manuscript. SB and J-SZ supervised the study and provided the funding. All authors approve it for publication.

### Conflict of Interest Statement

The authors declare that the research was conducted in the absence of any commercial or financial relationships that could be construed as a potential conflict of interest.

## Abbreviations

AER: Apical ectodermal ridge
PZ: Progress zone
ZPA: Zone of polarizing activity
Fgf10: Fbiroblast growth factor
Fgfr2b: Fibroblast growth factor receptor 2b
Tbx: T-box
Hox: Homebox
Etv: E26 transformation-specific translocation variant.
